# Supplementary figures and images for: Identification and Analysis of a Four-Gene Set for Diagnosing SFTS Virus Infection Based on Machine Learning Methods and Its Association with Immune Cell Infiltration
Source: Viruses. 2023 Oct 20;15(10):2126. doi: 10.3390/v15102126 (PMC10612101; doi:10.3390/v15102126)

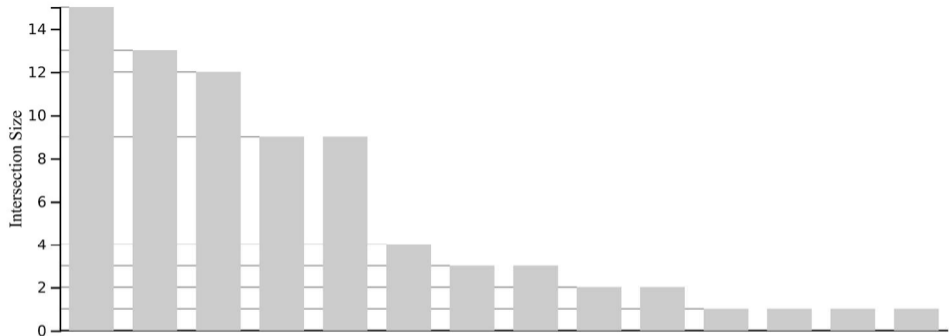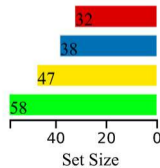

ADIPOR1

CENPO

H2AC17

E2F2

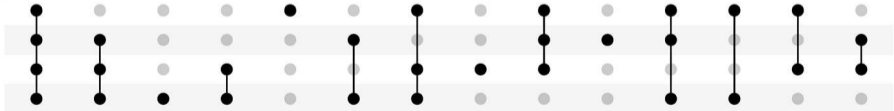

Supplement: Supplementary file 1 [file viruses-15-02126-s001.zip › Figure S1.pdf]
